# *Notes from the Field: *SARS-CoV-2 Omicron Variant Infection in 10 Persons Within 90 Days of Previous SARS-CoV-2 Delta Variant Infection — Four States, October 2021–January 2022

**DOI:** 10.15585/mmwr.mm7114a2

**Published:** 2022-04-08

**Authors:** Mellisa Roskosky, Brian F. Borah, Peter M. DeJonge, Catherine V. Donovan, Lynn Zanardi Blevins, Allison G. Lafferty, Julia C. Pringle, Patsy Kelso, Jonathan L. Temte, Emily Temte, Shari Barlow, Maureen Goss, Amra Uzicanin, Allen Bateman, Kelsey Florek, Vance Kawakami, James Lewis, Julie Loughran, Sargis Pogosjans, Meagan Kay, Jeff Duchin, Stephanie Lunn, Hannah Schnitzler, Shivani Arora, Jacqueline Tate, Jessica Ricaldi, Hannah Kirking

**Affiliations:** ^1^Epidemic Intelligence Service, CDC; ^2^Public Health – Seattle & King County, Seattle, Washington; ^3^Vermont Department of Health; ^4^Wisconsin Department of Health Services; ^5^Rhode Island Department of Health; ^6^Career Epidemiology Field Officer Training Program, CDC; ^7^University of Wisconsin, Madison, Wisconsin; ^8^National Center for Emerging and Zoonotic Infectious Diseases, CDC; ^9^Wisconsin State Laboratory of Hygiene, Madison, Wisconsin; ^10^Washington State Department of Health; ^11^CDC COVID-19 Emergency Response Team.

Vaccination protects against infection with SARS-CoV-2 (the virus that causes COVID-19) and related hospitalizations (*1*,*2*), and surviving a previous infection protects against B.1.1.7 (Alpha) and B.1.617.2 (Delta) variant reinfections^†^ (*2*). Since the SARS-CoV-2 B.1.1.529 (Omicron) variant became predominant in the United States in late December 2021, reported reinfections have increased^§^ (*3*). Early reinfections (those occurring within 90 days of previous infection) are not well understood (*4*). Because some persons have prolonged detection of viral RNA after infection,^¶^ repeat positive nucleic acid amplification test (NAAT) results within 90 days could reflect prolonged shedding from earlier infection, presenting technical challenges to documenting and characterizing early reinfections. This report describes 10 patients from four states, with whole genome sequencing (WGS)–confirmed Omicron variant infections within 90 days of a previous Delta infection. This activity was reviewed by CDC, approved by respective institutional review boards, and was conducted consistent with applicable federal law and CDC policy.**

An early reinfection was defined as a SARS-CoV-2 WGS test result (performed at a state, university, or contracted commercial laboratory^††^) from a new NAAT-positive specimen, collected during October 2021–January 2022 and <90 days after a first positive specimen from a previous WGS-confirmed SARS-CoV-2 infection, that demonstrated a different lineage from the first infection. Vermont Department of Health case investigators noted an increase in suspected early reinfections; five of these cases were confirmed through Vermont’s passive WGS surveillance system, which sequences the highest percentage (15.8%) of total state cases nationwide.^§§^ Wisconsin Department of Health Services was notified by university researchers of suspected early reinfections in members of a household enrolled in a longitudinal respiratory disease surveillance study.^¶¶^ Public Health – Seattle & King County was notified after Washington testing guidance for K–12 schools led to identification of a suspected early reinfection in a student at a school sporting event. Rhode Island screening protocols for hospitals and long-term care facilities led to collection of two NAAT-positive specimens within 90 days from a long-term care facility resident.

Ten patients with early reinfections were identified (Table). WGS identified Delta variant in all specimens from first infections and Omicron in all reinfection specimens.*** Median age at first infection was 11 years. Eight patients were aged <18 years, one was a long-term care facility resident, and one was a health care worker^†††^; five were male. Intervals between initial and subsequent specimen collections ranged from 23 to 87 days (median = 54.5 days). Patient E had completed a 2-dose mRNA COVID-19 vaccination series 6–10 weeks before the first infection; patients A and B each had received a single dose of mRNA COVID-19 vaccine between infections. The seven remaining patients were unvaccinated. In Wisconsin, household transmission during patient G’s reinfection likely resulted in reinfections of patients F and H.^§§§^^,^^¶¶¶^ Nine patients were symptomatic during first infection (median duration = 9 days; range = 0–20 days).**** Among eight patients with available clinical data during reinfection, six were symptomatic during reinfection (median duration = 5 days; range = 0–10 days).

**TABLE:** Characteristics of SARS-CoV-2 Omicron variant infection in 10 persons within 90 days of a previous SARS-CoV-2 B.1.617.2 (Delta) variant infection — four states, October 2021–January 2022

Patient	State	Age group, yrs*	Race and ethnicity	High-risk preexisting condition^†^	Infection no.^§^	Test date	COVID-19 vaccination status	Suspected exposure	Symptoms	No. of days between infections
A	Vermont	5–11	White, NH	No	1	Oct 19, 2021	None	School	Yes	87
					2	Jan 14, 2022	1 mRNA dose (Dec 17, 2021)	Household	Yes	
B	Vermont	5–11	White, NH	No	1	Oct 30, 2021	None	School	Yes	77
					2	Jan 15, 2022	1 mRNA dose (Jan 8, 2022)	Family gathering	Yes	
C	Vermont	5–11	White, NH	Yes	1	Nov 21, 2021	None	Household	Yes	69
					2	Jan 29, 2022	None	Household	Yes	
D	Vermont	0–4	White, NH	No	1	Nov 11, 2021	None	School	Yes	76
					2	Jan 26, 2022	None	Unknown	Unknown	
E	Vermont	25–39	Black, NH	Yes	1	Dec 16, 2021	2 mRNA doses (Sep/Oct 2021)	Health care	Yes (hospitalized)	40
					2	Jan 25, 2022	(As above)	Health care	No	
F^¶^	Wisconsin	5–11	White, NH	No	1	Nov 27, 2021	None	School	Yes	45
					2	Jan 11, 2022	None	Household (patient G)	Yes	
G^¶^	Wisconsin	5–11	White, NH	No	1	Dec 4, 2021	None	Household (patient F)	Yes	31
					2	Jan 4, 2022	None	Unknown	Yes	
H^¶^	Wisconsin	5–11	White, NH	No	1	Nov 27, 2021	None	Household (patient F)	Yes	52
					2	Jan 18, 2022	None	Household (patient G)	Yes	
I	Washington	12–17	White, NH	No	1	Nov 23, 2021	None	Household	Yes	23
					2	Dec 16, 2021	None	School sport	No	
J	Rhode Island	65–74	Unknown	Unknown	1	Nov 15, 2021	None	LTCF	No	57
					2	Jan 11, 2022	None	LTCF	Unknown	

**Abbreviations:** LTCF = long-term care facility; NH = non-Hispanic.

* At time of first infection.

^†^ Obesity, diabetes mellitus, chronic kidney disease, chronic obstructive pulmonary disease and bronchiectasis, neurocognitive disorders, coronary arteriosclerosis, and other heart disease.

^§^ In all cases, the first infection was with Delta variant and the second was with Omicron variant.

^¶^ Patients were in one household.

Expansion of SARS-CoV-2 WGS, through public health surveillance and longitudinal research,^††††^ might enable rapid identification of reinfections with distinct lineages and detection of novel variants. Current CDC guidance for identifying early reinfections requires demonstration of different lineages by genetic sequencing.^§§§§^ Limited capacity for strain testing, including WGS, diminishes opportunities for first and reinfection NAAT specimens from the same person to undergo additional testing.^¶¶¶¶^ Moreover, antigen tests are increasingly performed at home, resulting in specimens being unavailable for strain testing. Thus, most early reinfections are likely not identified.

The findings from this case series might not be generalizable to the U.S. population and are specific to the transition period between Delta and Omicron variant predominance. Nonetheless, this study highlights potential limits of infection-induced immunity against novel variants.

One patient in this case series had received a full primary COVID-19 vaccine series but was not yet eligible for a booster. No other eligible patient was up to date on recommended COVID-19 vaccinations,***** which provides additional protection, even among those with previous infection (*2*,*5*). These patients might have had increased risk for SARS-CoV-2 infection because of low vaccination rates^†††††^ and high rates of close contact^§§§§§^ in school-aged cohorts, and higher frequency and intensity of exposures in health care and congregate settings. Although the epidemiology of COVID-19 might change as new variants emerge, vaccination remains the safest strategy for preventing future SARS-CoV-2 infections (*2*,*5*).

## Footnotes

†
https://www.cdc.gov/coronavirus/2019-ncov/science/science-briefs/vaccine-induced-immunity.html

§
https://coronavirus.health.ny.gov/covid-19-reinfection-data

¶
https://www.cdc.gov/coronavirus/2019-ncov/hcp/duration-isolation.html

** Inclusion of Wisconsin case data was approved by University of Wisconsin-Madison, Minimal Risk Research Institutional Review Board; 45 C.F.R. part 46; 21 C.F.R. part 56. Remaining activity was determined not to be research; 45 C.F.R. part 46, 21 C.F.R. part 56; 42 U.S.C. Sect. 241(d); 5 U.S.C. Sect. 552a; 44 U.S.C. Sect. 3501 et seq.

†† Despite potential differences in established WGS protocols among laboratories, all lineage and sublineage determinations, which are based upon universally accepted genetic sequence markers, are comparable among laboratories.

§§ In the 90 days preceding April 4, 2022, the percentage of state cases sequenced and shared with GISAID ranged from 0.45% (Oklahoma) to 15.83% (Vermont). https://www.gisaid.org/submission-tracker-usa

¶¶
https://www.fammed.wisc.edu/orchards

*** Multiple Delta variant sublineages (AY.3, AY.4, AY.25, AY.33, AY.44, AY.47, and AY.100) and two Omicron variant sublineages (BA.1 and BA1.1) were identified.

††† Patient E was a non-Vermont resident who was living and working as a health care worker (caring for COVID-19 patients) in Vermont during both infections.

§§§ The index member in this household cluster of persons with early reinfections with SARS-CoV-2 was patient G, who began to experience respiratory illness symptoms on January 3, 2022, and was the only person to receive a positive SARS-CoV-2 test result the next day when specimens were collected from all household members. On January 8, patient F began to experience respiratory illness symptoms and was the only person to receive a positive SARS-CoV-2 test result when specimens from remaining household members (including patient G) were tested on January 11. On January 15, patient H began to experience symptoms of respiratory illness and was the only person to receive a positive SARS-CoV-2 test result when specimens from remaining household members (including patients F and G) were tested on January 18.

¶¶¶ Household secondary transmission during patient B’s early reinfection likely also resulted in an early reinfection in a parent, but WGS data from this parent’s reinfection were not available for confirmation. This parent is not included in this case series.

**** Calculation of median duration of symptoms during first infection does not include patient D because information on the full duration of this patient’s symptoms was unavailable.

†††† The Seattle Flu Study, like the Oregon Child Absentee due to Respiratory Disease Study (ORCHARDS), is a community-based, longitudinal surveillance study of influenza and viral respiratory diseases. Seattle Flu Study researchers were instrumental in the first identification of COVID-19 in the Seattle area. http://www.seattleflu.org/

§§§§
https://ndc.services.cdc.gov/case-definitions/coronavirus-disease-2019-2021/

¶¶¶¶ Although S-gene target failure, which detects a deletion in the gene that encodes for the SARS-CoV-2 spike protein, is a commonly used screening method for the Omicron variant, this deletion is not unique to this variant and is not present in all Omicron variant sublineages. Other strain testing methods only target a portion of a strain’s genome. In contrast, WGS analyzes a strain’s entire genome and is therefore the preferred method for lineage confirmation.

*****
https://www.cdc.gov/coronavirus/2019-ncov/vaccines/stay-up-to-date.html

†††††
https://covid.cdc.gov/covid-data-tracker/#vaccination-demographics-trends

§§§§§
https://www.nature.com/articles/s41598-021-81673-y
